# Personal Care Products as a Contributing Factor to Antimicrobial Resistance: Current State and Novel Approach to Investigation

**DOI:** 10.3390/antibiotics12040724

**Published:** 2023-04-07

**Authors:** Giulia Caioni, Elisabetta Benedetti, Monia Perugini, Michele Amorena, Carmine Merola

**Affiliations:** 1 Department of Life, Health and Environmental Sciences, University of L’Aquila, 67100 L’Aquila, Italy; elisabetta.benedetti@univaq.it; 2 Department of Bioscience and Technology for Food, Agriculture and Environment, University of Teramo, Via Balzarini 1, 64100 Teramo, Italy; mperugini@unite.it (M.P.); mamorena@unite.it (M.A.); cmerola@unite.it (C.M.)

**Keywords:** antimicrobial resistance, personal care products, additives, parabens, triclosan, triclocarban, zebrafish, artificial intelligence, machine learning

## Abstract

Antimicrobial resistance (AMR) is one of the world’s industrialized nations’ biggest issues. It has a significant influence on the ecosystem and negatively affects human health. The overuse of antibiotics in the healthcare and agri-food industries has historically been defined as a leading factor, although the use of antimicrobial-containing personal care products plays a significant role in the spread of AMR. Lotions, creams, shampoos, soaps, shower gels, toothpaste, fragrances, and other items are used for everyday grooming and hygiene. However, in addition to the primary ingredients, additives are included to help preserve the product by lowering its microbial load and provide disinfection properties. These same substances are released into the environment, escaping traditional wastewater treatment methods and remaining in ecosystems where they contact microbial communities and promote the spread of resistance. The study of antimicrobial compounds, which are often solely researched from a toxicological point of view, must be resumed considering the recent discoveries, to highlight their contribution to AMR. Parabens, triclocarban, and triclosan are among the most worrying chemicals. To investigate this issue, more effective models must be chosen. Among them, zebrafish is a crucial study system because it allows for the assessment of both the risks associated with exposure to these substances as well as environmental monitoring. Furthermore, artificial intelligence-based computer systems are useful in simplifying the handling of antibiotic resistance data and speeding up drug discovery processes.

## 1. Introduction

One of the key concerns for industry is the capacity to offer items that are risk-free for consumers while also guaranteeing the preservation of their chemical, physical, and microbiological properties. When it comes to producing edible goods or products used for personal hygiene and cleaning, additives play a significant role in regulating the bacterial load of the items. Without them, the material would quickly expire, and, more importantly, it would put the consumer at risk of unpredictable dangers. Antimicrobial agents are also added in the formulation of medical lotions, soap, creams, or sprays, used for everyday hygiene or the treatment of localized bacterial infections. Some of the most well-known chemicals include parabens, triclocarban, and triclosan. Starting in the mid-1920s, parabens (*p*-hydroxybenzoic acid esters) were commonly added to cosmetics, food products, and pharmaceuticals; their wide use was related to their low costs and good stability [1]. Triclocarban and triclosan can be found in several personal care products, such as toothpaste, detergents, shampoos, deodorants, and body washes [2]. These substances are considered contaminants of emerging concern because of their occurrence in outdoor and even indoor environments [3,4,5]. Their persistence in several environmental matrices is also the consequence of incomplete elimination by the traditional wastewater treatment process. For instance, analysis performed on effluent samples from wastewater treatment plants showed the presence of high paraben residuals. The most predominant form that was detected was methylparaben, which reached a concentration of 3830 ng/L in United States effluent before 2012 [6]. Parabens were also found in sediments and sludge samples that were collected in Japan and Korea [7].

Triclosan and triclocarban were also found at effluent discharge sites in the United States, Australia, Switzerland, Japan, and China, with concentrations ranging from 10.9 to 241 ng/L for triclosan and 23.9 to 342 ng/L for triclocarban, respectively [2,8,9,10,11,12]. The contamination is aggravated by these compounds’ low solubility, which causes them to accumulate and persist in surface waters and sediments. Surface sediments obtained from northern China’s coastal areas contained at least one of the primary paraben analogs, with values ranging from 1.37 to 24.2 ng/g (dry weight) [13]. The supplementary research involved sediment investigations from various Chinese rivers [14,15], as well as lakes and bays in America and Spain [7,16,17]. However, the issue of accumulation impacts not just terrestrial or aquatic ecosystems, but also the residential environment which serves as an extra storage location for harmful chemicals. An investigation of 80 samples of U.S. indoor dust gathered from residential dwellings and sporting facilities, for example, revealed the presence of antimicrobial compounds such as paraben esters (methyl, butyl, ethyl, benzyl paraben), triclocarban, and triclosan [18].

Several studies have demonstrated the role of these chemicals as possible endocrine disruptors [19,20]. Concerns have been expressed regarding how these substances may impair organism development, particularly in cases of indirect exposure, such as that experienced by pregnant women. Much research on zebrafish has revealed that the developmental processes of early life stages can be affected depending on the concentration examined [21,22,23]. The impacts on development have also been examined on human communities, and several findings have fueled worry [24,25,26].

### Usage of Personal Care Products and the Spread of Infectious Diseases

Antimicrobial substances prevent the growth of germs through various mechanisms. The potential for choosing resistant strains indicates the dangers connected to the excessive use of these agents [27,28,29,30,31]. AMR, for example, is a concern in the treatment of *Mycobacterium tuberculosis* infections, since it limits the number of available antibiotics for its therapy [32]. The same goes for infections caused by *Clostridium difficile*, which continue to be a severe danger and burden for healthcare systems [33].

Antibiotic overuse in the treatment of infections, particularly in children, is historically linked to the issue of antimicrobial resistance (AMR) [34,35], but also the usage of personal care products plays a key role. These compounds are discharged into the environment, and due to their impact on microbial ecosystems, their persistence dictates when resistance phenomena start to occur. Due to their relatively high concentrations, these chemicals exert selective pressure on the microbial populations that inhabit both domestic and natural habitats [36].

In particular, the spread of more or less hazardous diseases has been encouraged by high cleanliness standards. The use of personal care products is directly tied to this paradox. As hands are a major source of bacterial and viral transmission, handwashing is critical in reducing the risk of infection, and the use of antibacterial soaps has been demonstrated in studies to help prevent cutaneous infections and treat skin lesions [37]. Correct hand cleansing may be effective in reducing bacterial illnesses such as gastrointestinal illness, but the additives contained in soaps may contribute to the spread of resistance. For instance, exposure to triclosan resulted in increased resistance to chloramphenicol and tetracycline in *Salmonella enterica* serovar Typhimurium [38] and *Escherichia coli* [39].

Many infectious disease symptoms were much more common in people with poor health or chronic problems who used antibacterial medications [40], signaling that more research is needed. The effects of antibiotic resistance on respiratory system health warrant special consideration [41]. Bacteria that are specifically selected by antimicrobial agents cause airway infections. *Pseudomonas aeruginosa*, for example, is responsible for lung infections and is resistant to triclosan at high concentrations [42]. Parabens are not effective against *Staphylococcus aureus*, the causative agent of staphylococcal pneumonia [43]. These are only a few of the hazards connected with utilizing antimicrobials that are detrimental both on their own and in combination with other antibiotics.

This review aims to look at any connections that might exist between using personal care products and developing AMR. We shall pay particular attention to three additives whose widespread use in a variety of preparations has prompted severe environmental concerns (parabens, triclocarban, and triclosan). Given the need to investigate these substances from a novel perspective, new experimental models must be developed to facilitate the collection of new data. Understanding these challenges is a critical step in creating awareness for the preservation of people and the environment.

## 2. Parabens, Triclocarban, and Triclosan: The Dark Side of Personal Care Products

Parabens, triclocarban, and triclosan are well-known chemicals found in a variety of products. Parabens are preservatives that are added to a variety of items to strengthen their stability over time, such as cosmetics, detergents, or lotions. They are derived from benzoic acid and comprise derivatives characterized by the different lengths of the side chain. Its excessive use has resulted in its release into the environment, where it has contaminated several matrices. Its presence in the environment also influenced microbial ecosystems, leading to the selection of resistant strains. Selvaraj and colleagues (2013) investigated the resistance of bacteria in sewage treatment plant effluents to parabens and discovered that *S. aureus* was the most resistant while *P. aeruginosa* is the least resistant. Butyl and ethyl parabens are often more toxic to bacteria [44]. These studies suggested that environmental paraben exposure could contribute to the development of resistance in pathogenic bacteria, leading to detrimental consequences for human health. Nonetheless, there is also concern that these chemicals, once swallowed, may interact with the microbial communities of the human mucous membranes. Even though there is relatively little research, investigations on rats yield fascinating outcomes. For example, low dose methylparaben exposure could influence bacterial composition changes in rats during their adolescence [45]. This finding is critical since gut dysbiosis may be one of the mechanisms permitting multidrug-resistant pathogenic bacteria to colonize the intestinal mucosa [46].

Triclocarban and triclosan are broad-spectrum antibacterial compounds that have found widespread use in a variety of cosmetic products including skin disinfectants and sanitizers. However, because of concerns about their impact on hormones and antibiotic resistance, regulatory bodies such as the FDA and the European Commission have prohibited the use of triclocarban and triclosan in some commodities. Despite this, they are still widely utilized in many consumer products, and once in the environment, these chemicals can be found in water, sediment, aquatic animals, dust, and even human bodies [47]. The presence of triclosan in sewage sludge makes it an important source of antibiotic resistance genes, and mutations in the enoyl-acyl carrier protein (ACP) reductase gene have been discovered in sensitive opportunistic bacteria exposed to triclosan [48]. Triclocarban exposure in sewage sludge can reduce antibiotic tolerance and promote the selection of cross-resistant and multidrug-resistant genes, helping ARGs and MGEs (antibiotic resistance genes and mobile genetic elements) propagate in bacterial communities [49,50].

To acquire a more complete picture, these compounds should not only be studied separately, but also in the context of combination toxicity [51,52]. The environment contains a mix of chemical contaminants, including pharmaceuticals and personal care products, industrial chemicals, nutrients derived from wastewater and manure, and dissolved organic matter. Wastewater treatment plants release these contaminants into rivers, and their quantities can vary depending on social and economic factors. For example, the COVID-19 pandemic has led to increased use of antibacterial substances and disinfectants, certainly impacting the environmental microbial communities [53].

The main properties and characteristics of parabens, triclocarban, and triclosan are summarized in Table 1. Even though they have long been considered safe products, studies have increased to date that warn of the potential risks [54,55,56,57,58,59], with the emergence of AMR being a major concern.

### The Mechanisms of Antibiotic Resistance

Numerous studies have shown a connection between the usage of these compounds and the advent of AMR. For example, their impact on the microbial diversity of indoor environments was particularly well-expressed in a study by Fahimipour and colleagues (2018) [67]. Intriguingly, the study revealed that the abundance of microbial taxa with various resistance capacities is reflected in the presence of these chemicals in the household environment. They noticed the rise of phenotypes with cross-resistance to clarithromycin, ampicillin, or tetracycline [67]. These results supported the findings of Hartmann et al. (2016), who investigated the connection between the dust microbiome and the antibacterial substances triclosan, triclocarban, and paraben derivatives [68]. The analysis of dust resistome revealed a high abundance of diverse genes of resistance such as *tet(W)*, *blaSRT-1*, and *erm(B)* [68]. They performed different analyses on 44 dust samples collected in an athletic and educational facility, showing a positive correlation between the concentrations of antimicrobial agents and the abundance of resistome (the collection of genes responsible for antibiotic resistance) [68].

The dust accumulated in homes or other buildings represents a suitable substrate for this kind of examination. It contains a sizable portion of biological components, such as microbes. Since indoor communities are strongly influenced by anthropogenic and domestic factors (temperature, humidity, diet, habits, and hygiene) [69], antimicrobial substances, dust quality, and an increase in antibiotic genes might be connected. Other surveys were carried out on dust samples from 63 Canadian houses, 10 private residences in the northwest of Spain, and 18 houses in Belgium, demonstrating that indoor dust may act as a sink of antimicrobial compounds, including the above-mentioned ones [70,71,72]. Dust contamination suggests that these substances may persist in the environment at home and continue to encounter people (especially children, who ingest them more easily).

In addition to the residential environment, these compounds continue to exist outdoors in many matrices, as was previously mentioned in the introduction. The main implication of their persistence is the selection of resistant bacterial communities. According to some studies, exposure to antimicrobials can favor the development of bacteria that are resistant to both biocides and antibiotics. This occurs because some of the genes that give resistance to biocides are found on the same genetic elements as those that provide resistance to antibiotics, and the use of biocides can encourage the spread of these genes among bacterial populations. For example, the presence of triclocarban and triclosan could influence the abundance of the *tet(Q)* gene, responsible for resistance to tetracycline, in aerobic-activated sludge microcosms [73]. The cross-resistance between triclosan and antibiotics in *P. aeruginosa* is another example. It had been proven that triclosan and antibiotic exposure might select for equivalent mutations that result in the expression of a multidrug efflux mechanism [74]. In addition, it has been demonstrated that exposure to environmentally relevant concentrations of triclosan could promote the conjugative transfer of multi-resistance genes across different bacterial genera, by the promotion of reactive oxygen species (ROS) generation [75].

**Figure 1:**
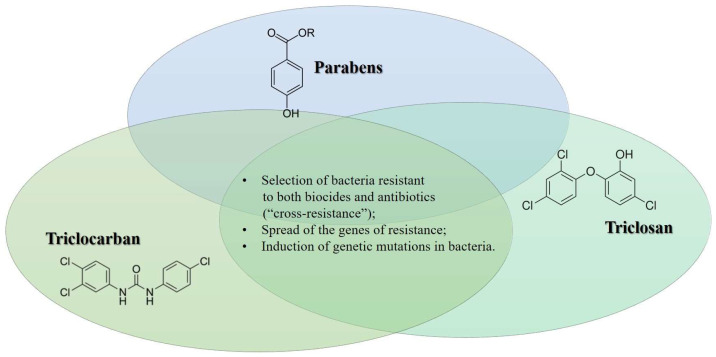
Biocide and the onset of antimicrobial resistance: summary of the main mechanisms.

Thus, it is evident that biocides may alter permeable bacterial cell walls, making it more challenging for antibiotics to kill germs by penetrating and entering the cells. In a 2005 study, Bredin and colleagues demonstrated that propylparaben can cause the release of potassium from *E. coli* cells via a permeabilizing impact, in which the porin OmpF appears to be involved [76]. As a result, this compound can select any Gram-negative bacteria with poor porin expression. Those same bacteria have been noted to exhibit a considerable level of β-lactam resistance [77]. In Figure 1, we summarize the main mechanisms of antimicrobial resistance mediated by the biocides reported above.

Research employing diverse models, ranging from cellular (or two-dimensional) to in vivo, has led to the understanding of resistance pathways, while epidemiological research has contributed significantly to our understanding of the trend and spread of AMR. It is critical to understand how antimicrobial resistance develops and spreads, as well as create measures to prevent and regulate its dissemination. This necessitates interdisciplinary study integrating microbiology, epidemiology, medicine, public health, and social sciences, among others. Through understanding antimicrobial resistance, we may create novel and effective antibiotics, adopt appropriate antibiotic use policies, and improve infection prevention and control strategies, ultimately leading to better health outcomes for individuals and communities.

## 3. Modeling and Tracking Antimicrobial Resistance: From Bi-Dimensional Models to Artificial Intelligence

The given examples highlight how AMR is an emerging worldwide health issue that is anticipated to get worse over time and eventually kill millions of people. According to the 2019 study on the worldwide burden of bacterial resistance, there were 4.95 million drug-resistant infection-related deaths, of which at least 1.27 million were directly attributable to drug resistance [78]. Therefore, the only factors that contribute to a greater death risk are ischemia and heart attacks. *E. coli*, *S. aureus*, *K. pneumoniae*, *S. pneumoniae*, and *P. aeruginosa* are a few of the key pathogens that are thought to be to blame for this rise in mortality.

However, the discovery of a significant link with antimicrobial compounds found in numerous widely used items makes it evident that more research into the mechanisms underlying antibiotic resistance is required, as well as the development of new tracking systems.

### 3.1. The Potentiality of the Zebrafish Model

**Figure 2:**
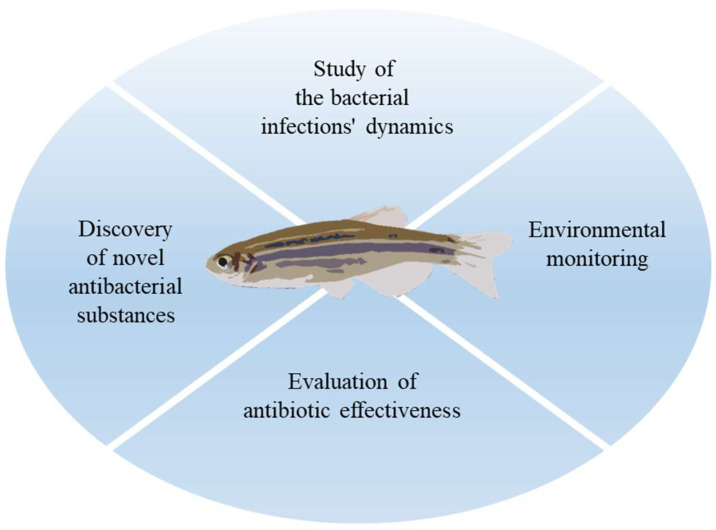
The use of zebrafish in the study of AMR.

Our understanding of the mechanisms underlying antibiotic resistance has surely been aided by the traditional method of researching how various chemicals affect bacterial cells directly [79,80,81,82]. The potential to research resistant infections using the zebrafish model is particularly intriguing because it already has many benefits and enables the investigation of numerous issues (Figure 2).

Given that it shares many metabolic, physiological, and developmental processes with humans, zebrafish can be utilized to research antibiotic resistance and the host’s response to bacterial infections [83]. New antibacterial substances can be tested for toxicity and effectiveness using zebrafish [84]. For instance, zebrafish can be exposed to various compound concentrations and several parameters such as survival, rate of development, and organ morphology could be monitored. Utilizing this method, one can find novel chemicals that are effective against particular strains of antibiotic-resistant bacteria [85].

The effects of antimicrobial drugs on development have been studied extensively in both embryonic and larval forms of zebrafish [21,23,86]. In this regard, it will undoubtedly make it possible to assess how antibiotic resistance impacts survival in infection cases and identify new targets to get around the problem of recurrent drug resistance. Zebrafish also serve as a model for environmental monitoring, making it feasible to simulate the effects of exposure to specific biocide doses while also allowing for the evaluation of the interaction with microbiota [87,88,89]. For instance, the study of bacterial species isolated from zebrafish itself raises intriguing questions, as demonstrated by a collection of 43 *Aeromonas* species that were isolated and recognized from distinct zebrafish specimens selected from various pet stores. The genes for antibiotic resistance have been recognized, and they have helped to establish the MDR (multidrug resistance) indexes [90].

The broad-spectrum antibiotic oxytetracycline (OTC) is an environmental polluter that is commonly used in aquaculture. The zebrafish model was utilized by Almeida and colleagues (2021) to examine the effects of chronic OTC exposure on the microbial population in the environment and the fish’s gut microbiota [91]. They discovered that OTC had a significant impact on the levels of particular bacteria after 5 days and 1 month of exposure [92]. The ability of an organism to recover at the microbiome level following chemical exposure is critical for assessing populations’ ability to recover from situations of intermittent or pulsed contamination [93].

Even more recently, the zebrafish model has proven to be especially useful in assessing how chlorine residues affect the spread of AMR. It is a quite relevant topic since the usage of chlorine-based disinfection products increased during the COVID-19 pandemic. The research showed that freshwater microbial communities can be disturbed by chlorine, even at modest levels, when it is introduced continuously [94]. This may lead to a decrease in their metabolic rate, an increase in potential pathogens, and an acceleration of the transmission of antibiotic resistance genes (ARGs). Zebrafish’s intestinal microbial population is also adversely impacted, which harms their growth and behavior. According to the study, microbial diversity and richness were reduced by exposure to 0.1 mg/L of chlorine, but they soon recovered. After 7 days of exposure, chlorine also had an impact on the majority of microbial species, but by 14 days, the effects had been reversed [94].

### 3.2. Artificial Intelligence against Antimicrobial Resistance

Traditional research offers a wealth of knowledge regarding the molecular pathways underlying antibiotic resistance, and even experiments carried out directly on sick animals give us a more comprehensive picture. However, new technologies play a significant part in monitoring antibiotic resistance. There may be several options to explore antibiotic resistance thanks to artificial intelligence (AI) [95]. AI may be used, for instance, to analyze vast volumes of data that are produced by studies on antibiotic resistance, spot trends, and make predictions about the spread of antibiotic resistance. AI can also be used to find new therapeutic targets and enhance the identification of new antibiotics. Large datasets of chemical compounds can be analyzed using machine learning algorithms to forecast their qualities, such as their capacity to combat a particular pathogenic bacterium or prevent antibiotic resistance [96]. AI can also help make the use of antibiotics more effective. For instance, patient response to a specific antibiotic can be predicted using machine learning algorithms, customizing treatment, and enhancing clinical efficacy [97]. AI enables health authorities to promptly implement preventive measures by predicting and tracking the spread of illnesses that are resistant to antibiotics in real-time [98]. AI can make a significant contribution to the fight against antibiotic resistance by helping with drug discovery, infection prevention and control, and patient-specific antibiotic treatment [99].

Machine learning, a subfield of artificial intelligence, has emerged as a viable way for dealing with this complexity by constructing algorithms capable of predicting outcomes with minimal human intervention. It can be used to predict resistance phenotypes from pathogen genomic data, to better understand antibiotic processes and medication development, and to aid in antimicrobial stewardship in clinical decision-making. While the area is still in its early stages, machine learning is expected to play a significant role in lowering the global burden of antimicrobial resistance [100]. By accelerating target identification, lead discovery, preclinical and clinical development, machine learning has the potential to revolutionize pharmaceutical drug discovery. Recent studies have shown that machine learning can be applied to the production of novel antimicrobials by learning small molecule structural features from existing screens in the context of antimicrobial development [101]. This advancement has included both novel screening procedures and new algorithms that increase algorithmic learning by utilizing chemical structure representations. Johnson et al. (2019), for example, used a genomic library and machine learning to uncover new therapeutic targets to establish a screening technique for discovering pharmacologic inhibitors of key genes in *Mycobacterium tuberculosis* [102].

## 4. Conclusions

Antibiotic resistance is a problem of global importance with repercussions on human health. Personal care products and, in particular, the antimicrobial additives they contain, are released into the environment and meet microbial communities, altering them and selecting resistant bacteria. These substances have also been detected in indoor environments, further increasing the concern about their persistence. We concentrated on three of these additives because they are found in many personal care products: parabens, triclosan, and triclocarban. Although legislation in this area has resulted in a reduction in the percentages of presence allowed in marketed products over time, they are still found in ecosystems due to their proclivity to persist. Therefore, it becomes necessary to research and examine how these compounds may affect microbial ecosystems and result in AMR. To accomplish this goal, new analytical approaches are also required to highlight the AMR processes and, more importantly, to find novel biological targets to solve the issue of resistance. We have concentrated on the use of zebrafish as an alternative to conventional models since it plays a crucial part in monitoring harmful compounds that are released into the environment. The monitoring of antibiotic resistance can also be aided by artificial intelligence and machine learning approaches which can enable the research of this issue through simulations and in silico models.

## Figures and Tables

**Table 1:** Main characteristics of parabens, triclocarban, and triclosan.

Agents	Sources	Main Characteristics
Parabens (p-Hydroxybenzoates derivatives)	Cosmetics (makeup, moisturizers, hair care, and shaving products), food, drinks, and pharmaceuticals [60].	Broad spectrum of antimicrobial activity, chemical stability, and low production costs.
		*Physical properties*: small colorless crystals or crystalline powders (no odor, no taste).
		*Solubility*: high oil/water coefficient.
		*Stability*: they are stable in air, in hot or cold water, and in acidic solutions [1].
		*Mechanism of action:* According to some theories, they inhibit the synthesis of DNA and RNA [61], block the activity of some crucial enzymes, such as ATPases and phosphotransferases in some bacterial species [62], or interfere with membrane transport procedures [63]. The antimicrobial properties increase with increasing alkyl chain length.
Triclocarban	Antibacterial soaps, detergents, toothpaste, and body washes [64].	It is a triclosan analog, and particularly effective against Gram-positive bacteria such as *Staphylococcus aureus* [64].
		*Physical appearance:* white plates or white powder, with a distinctive odor.
		*Mechanism of action:* it works by stopping the enzyme enoyl-acyl-carrier-protein (ACP) reductase from catalyzing the last step in each cycle of fatty acid elongation in the type II fatty acid synthase systems.
Triclosan	Dental care products, soaps, and cosmetics [56].	It is a broad-spectrum antimicrobial agent, effective against most Gram-negative and Gram-positive bacteria.
		*Physical appearance:* colorless to off-white crystalline powder (no taste, slightly aromatic odor).
		*Solubility:* highly soluble in organic solutions (benzene, ethanol, acetone) [65].
		*Mechanism of action:* it works by inhibiting enoyl-acyl carrier protein (ACP) reductase (FabI) [66].

## Data Availability

No new data were created or analyzed in this study. Data sharing is not applicable to this article.
